# “There’s an Etiquette to Zoom That’s Not Really Present In-Person”: A Qualitative Study Showing How the Mute Button Shapes Virtual Postpartum Support for New Parents

**DOI:** 10.1177/10497323231187541

**Published:** 2023-08-09

**Authors:** Anna MacLeod, Megan Aston, Sheri Price, Kathryn Stone, Rachel Ollivier, Britney Benoit, Meaghan Sim, Lenora Marcellus, Susan Jack, Phillip Joy, Masoumeh Gholampourch, Damilola Iduye

**Affiliations:** 1 3688 Dalhousie University, Halifax, NS, Canada; 2 Queen’s University, Kingston, ON, Canada; 3 1270 St. Francis Xavier University, Antigonish, NS, Canada; 4 432234 Nova Scotia Health, Halifax, NS, Canada; 5 8205 University of Victoria, Victoria, BC, Canada; 6 3710 McMaster University, Hamilton, ON, Canada; 7 3684 Mount Saint Vincent University, Halifax, NS, Canada

**Keywords:** virtual, postpartum, parents, mothers, mute button, Zoom

## Abstract

Virtual spaces that allow parents in the postpartum period to connect, support each other, and exchange information have been increasing in popularity. With the COVID-19 pandemic, many parents had to rely on virtual platforms as a primary means to connect with others and attend to their postpartum health. This study explored virtual postpartum support sessions through the web-based videoconferencing software, Zoom. Guided by feminist poststructuralism and sociomaterialism, we held seven virtual support sessions for parents caring for a baby 0–12 months in age, in Canada, and interviewed 19 participants about their experiences in the sessions. Our methodological approach allowed us to analyze discourses of (1) parenthood, (2) material realities of virtual environments, and (3) support and information on this virtual platform. The purpose of this research was to understand how technology influences postpartum support and learning through online videoconferencing for parents. Our findings document an overarching discourse of Zoom etiquette by which muting was a discursive practice that all participants used. The consistent use of the mute button while not talking structured conversation in virtual postpartum sessions and resulted in three themes: (1) minimizing disruptions; (2) taking turns; and (3) staying on task. The norm of using the mute button changed how parents received and gave support and information. Based on findings and broader literature, we discuss considerations for facilitation of virtual postpartum support sessions.

## Introduction

The transition to parenthood brings about a myriad of emotions. For many families, this is an exciting and positive experience; however, for some parents, caring for a baby and themselves during the postpartum period may be stressful and filled with complex psycho-social and physical changes (Aston et al., 2014, 2018). Most parents welcome postpartum education, support, and information to help them cope with the challenges and uncertainties of caring for a newborn (Price et al., 2018). Parents may choose to find support and information through in-person interactions with family, friends, health professionals, community workers, and drop-in centers, while others choose online sources including social media, chat spaces, blogs, and websites (Alang & Fomotar, 2015; Aston et al., 2021; Denton et al., 2020). Studies reveal that these virtual options are increasing in popularity and provide many benefits to parents, such as empowerment, confidence, and socialization (Aston et al., 2021; Denton et al., 2020; Stana & Miller, 2019; Valtchanov et al., 2014); however, prior to the COVID-19 pandemic, virtual supports were only one option of many. With the introduction of public health measures to mitigate viral transmission, many hospital and community health programs shifted to virtual modes of connection, significantly changing how parents receive help (Jackson et al., 2021; Wang et al., 2022). These digital connections were, for many, the primary connection to people outside their homes.

For years, socially constructed isolation during the postpartum period has been a documented challenge (Nowland et al., 2021). Mothers in particular report feelings of isolation and loneliness when transitioning to parenthood (Lee et al., 2019; McLaren, 2018; Taylor et al., 2021), which can increase the risk of developing moderate to severe mental health challenges including postpartum blues or depression (Boudiaf et al., 2022). Public health measures aimed at minimizing the spread of COVID-19 have compounded feelings of isolation and loneliness during the postpartum period (Gilbert, 2021; Joy et al., 2020; Kynø et al., 2021; Ollivier et al., 2021). However, there is evidence that access to technologically mediated communication tools and online support groups may reduce isolation and stress for new mothers (Jago et al., 2020; Robinson et al., 2019). Yet, all studies to date have focused on asynchronous, chat-based software with no audio or visual components, such as parent Facebook groups and mommy forums (Alang & Fomotar, 2015; Aston et al., 2021; Valtchanov et al., 2014).

### Videoconferencing Technologies

While videoconferencing software was used to connect virtually prior to the pandemic (Archibald et al., 2019), there was a significant uptake of these technologies starting in March 2020. The web-based videoconferencing software, Zoom, has particularly grown in use since the pandemic, becoming the most downloaded app in the Apple App Store in 2020 (Beauford, 2022). While other videoconferencing platforms like Microsoft Teams and Google Meet also grew in use, Zoom in particular became popular in healthcare fields because of its user friendliness and the availability of a free version (Domb et al., 2021; Wibowo et al., 2022). Zoom quickly became a primary means of connecting to others and even became part of daily vocabulary, for example, “Zoom fatigue” and “let’s Zoom.”

Along with the rapid uptake of videoconferencing platforms, an informal “etiquette,” or a code of polite behavior, developed which set parameters for how to use them. Discourses of “Zoom etiquette” suggest that people ought to ignore the technologies and behave as though one were in person (Zoom Communications, 2019), and this should be reflected in one’s appearance and behaviors while online (Boitnott, 2021). Most of this advice, however, disregards the agency and influence of videoconferencing technologies themselves. Far from neutral, cameras, screens, buttons, speakers, and microphones that facilitate connection actively influence the ways in which people participate (MacLeod et al., 2015). While they serve the purpose of allowing us to see and hear people who are geographically separate, they also make audible and visible things that we might not have had to contend with were we in person, such as remembering to click a button before speaking. Thus, mastering the mute button has become an essential skill, with the general consensus being that users should be muted while not speaking. This expectation is widely reinforced through an array of articles describing rules, tips, or “do’s and don’ts” for Zoom calls (Adams, 2020; Andersen, 2021; Boitnott, 2021).

Given the affordances of videoconferencing technologies to connect users to social support, resources, and services, Zoom and other similar platforms may be tools worth using in the post-pandemic era. Therefore, examining how parents used videoconferencing during the COVID-19 pandemic, and relatedly, how the tools of videoconferencing acted on and influenced new parents’ participation, is important. Such explorations can provide valuable information to understand how parents can be best supported online. The rapidly changing landscape of online videoconferencing, propelled by the COVID-19 pandemic, provides a unique opportunity to explore how the technologies of web-based videoconferencing are influencing a new generation of parents’ experiences of postpartum support and information.

### Study Purpose

The purpose of this research was to understand how technology influences postpartum support and learning through online videoconferencing for parents caring for a child 0–12 months in age. We used both feminist poststructuralism (FPS) and sociomaterialism to develop and explore the following questions: (1) How do parents, tools, and spaces come together and experience learning and support in videoconferenced environments? (2) How are these practices personally constructed within wider processes and social and institutional discourses of new parenthood?

## Methods

### Design

This study employed FPS and sociomaterial approaches to explore web-based videoconferencing (specifically Zoom), as a mechanism for offering postpartum virtual support (Aston, 2016; MacLeod et al., 2015). This layered theoretical approach allowed us to examine how support and learning in the postpartum period is interwoven with social and material factors. The complexities of social and material factors are heightened in a videoconference environment, where traditionally embodied educational and supportive interactions are heavily mediated by communication technologies. Using this approach, we were able to explore the personal, political, and social aspects of the postpartum experience while attuning to how these were brought forward through videoconferencing technologies.

FPS is both a theory and methodology (Cheek, 2000; Weedon, 1996), which enabled an exploration and examination of how new parents’ experiences accessing virtual postpartum supports were socially and institutionally constructed (Aston, 2016). FPS incorporates concepts such as agency, subjectivity, multiple ways of knowing, and relations of power (Weedon, 1987). The use of discourse analysis aided us in deconstructing the meaning of participant experiences by applying the concepts of beliefs, values, and practices (Aston, 2016; Foucault, 1982) to understand how dominant social and institutional discourses impacted their experiences. Further, FPS allowed us to examine and understand how parents used their agency to navigate relations of power within the virtual environment to challenge, accept, and question discourses related to the postpartum period for parents. In previous research conducted by the researchers (Aston and Price), FPS was applied to in-person gatherings, where we analyzed how discourses impacted how parents accessed information and support. Using FPS again enabled us to examine how support and information were uniquely accessed through the relatively new medium of videoconferencing technology.

While FPS provided an ideal approach to understand the discursive experience of online postpartum support and information, we were also interested in understanding how the materiality of the videoconferencing technologies influenced the discourse. We thus turned to sociomaterialism, which is an umbrella term for a set of theoretical perspectives suggesting that experience is brought about through assemblages of humans and non-humans (MacLeod & Ajjawi, 2020; MacLeod et al., 2015). Sociomaterialism decenters the human as the focus of study, encouraging us to consider the active role of the *things* in our environments—everything from internet connections to mute buttons. This allows for a deeper exploration of the complex and non-linear relationships between materials and social practices. The concept of symmetry is often employed, where human and non-human actors should be equally considered in analyses (MacLeod et al., 2015, 2019). As such, our sociomaterial orientation allowed us to attune to both the social and material elements of power and human interaction. Employing both sociomaterialism and FPS strengthened this study because it allowed for the consideration of individuals’ experiences negotiating social and institutional discourses while accounting for the intersection(s) of technology as part of everyday life.

### Academic Context

Our research team consists of a broad and diverse range of expertise, including critical qualitative methodologies, maternal and family health, clinical nursing, and sociomaterial perspectives. Our combined experience and expertise allowed us to develop a refined understanding of the interplay of discourse and materiality, leading to new insights about how the technologies of videoconferencing actively contribute to the postpartum experience. We approached reflexivity as an ongoing discussion throughout the study, engaging in purposeful reflective conversations as we planned the study, as well as while we collected, analyzed, and interpreted the data.

### Participants and Recruitment

This research was conducted in Nova Scotia, Canada, where COVID-19 distancing restrictions were in place at the time of this study. Participants were recruited through various means across Nova Scotia from October 2021 to April 2022. The recruitment poster was circulated on social media, in baby stores, at a lactation clinic, and on the postpartum unit of a hospital. Participants had to have been a parent or guardian to a baby between 0 and 12 months of age, live in Nova Scotia, and be able to connect online via Zoom to be eligible. We were interested in hearing multiple experiences and perspectives across Nova Scotia, so we did not set limiting restrictions other than being a parent and having access to the tools of videoconferencing.

### Virtual Postpartum Support Sessions

We conducted seven virtual postpartum support sessions between October 2021 and May 2022. Sessions had between four and eight participants per group, for a total of 37 participants. All support sessions were audio and visually recorded, and conducted by the primary investigator (PI), who has clinical experience as a public health nurse, and the research coordinator (RC). Discussion was guided by “Café Conversations,” which were conversation starters for new parent groups developed from previous research (mumsns.ca). Participants were made aware that these conversation starters were only a guide, and they could discuss anything they would like to. Participants were invited to share whatever they felt comfortable with and to use audio and video tools of Zoom as they wished. All sessions lasted 1 hour, and at the end of each session, the RC invited participants to email if interested in participating in an individual interview about their experiences.

### Data Collection

After each of the seven sessions, the PI and RC debriefed and wrote field notes regarding their immediate observations and experiences within the sessions. Nineteen individual, semi-structured interviews were conducted. Participants were asked about their experiences with the virtual support group broadly, followed by probes that focused on how they felt about their engagement in the session and how it made them feel. A focus on feelings and personal experiences enabled us to more closely examine participants’ beliefs and values about how a videoconferencing session addressed their postpartum needs and how they negotiated relations of power. Our questions were also geared toward uncovering sociomaterial aspects about their experiences such as how they decided to use their mute button, camera, chat space, etc., and how these tools made them feel. Questions were open-ended, for example, “Can you tell me about how you used your mute button?”. Participants were asked how things might have gone differently if the session had been conducted in person, which allowed researchers to explore important differences in the ways parents perceived support and information through a web-based videoconferencing platform. Interviews lasted approximately 30–60 minutes. Findings presented in this paper were drawn from the analysis of individual interviews, researcher’s field notes, and observations from session recordings. Following informed consent, demographic information was collected (see Table 1).

**Table 1.** Demographic Summary.

Age	Ranged from 28 to 41, with average of 32
Race and ethnicity	Two of African descent, one of Latin descent, one of Métis descent, one South Asian, one Mi’kmaq, 31 White or European ancestry
Gender	One non-binary, one preferred not to say, and 35 women
Sexual orientation	4 Queer, 33 heterosexual
Annual household income	Ranged from $80,000 to $210,000, with an average of $141, 813

### Ethical Considerations

Ethics approval was obtained from several units across Canada. Numbers were assigned to participants to protect their identities, and all identifying information was removed. All participants provided verbal consent prior to support sessions and interviews with the research coordinator.

### Analysis

FPS and discourse analysis, informed by sociomaterial theories, guided our analysis. The analysis was led by the principal investigator and the research coordinator, after which further analysis was performed by the other two co-investigators. This was followed by additional team members who contributed to analysis and writing. Our collective analysis focused on identifying and interpreting discursive practices and attending to how support and information were technologically mediated in the context of videoconferenced postpartum groups. This was an iterative and detailed process which involved first closely reading transcripts and then using a five-step process of FPS informed by discourse analysis (Aston, 2016). This allowed the team to first explore the words participants used to describe their personal beliefs, values, and practices pertaining to the virtual session. Next, the team explored the meaning of participants’ experiences by identifying different discourses pertaining to technologies and parenthood. This also included analyzing the way participants negotiated relations of power.

We next revisited the identified discourses to consider them with a sociomaterial lens. Sociomaterialism allowed us to explore how the material aspects of virtual postpartum support, including the ways people used and talked about the mute button, and how technological tools actively work to structure conversation, make people feel a certain way, or give people the option to act in certain ways. At the end of every transcript, the PI and RC created a list of notes focused on the active role of specific technologies, which were then integrated into final analysis. One of the co-investigators [lead author] with substantial expertise in sociomaterialism contributed extensively to analysis and led the team in this direction. Methodologies complimented each other and integrated well by virtue of their similar, critical framework of how experiences are constructed by the world around us. Findings presented in this paper represent one of five themes from our 2-year study titled the Virtual Village project.

## Findings

Engaging in Zoom etiquette was an overarching discourse in this study. The materiality of Zoom’s technologies required particular behaviors which, in turn, came to be considered normal, appropriate, and desirable. This structured the ways in which participants connected with one another. Although previous research has focused on the importance of video connection through cameras and screens, we noted that the ability to speak while on screen, mediated through the mute button, was not as fulsomely discussed. Yet, our data revealed that the mute button was a technology of consequence, and one that featured heavily in discourses of Zoom etiquette, and how people opted to participate.

Participants shared that etiquette for virtual interactions was different from etiquette for in-person interactions. Our analysis revealed that the mute button was critical, serving as an obligatory point of passage (Callon et al., 1986) which structured how people participated in Zoom sessions. We identified three distinct ways people used the mute button to reinscribe discourses of Zoom etiquette: (1) minimizing disruptions, (2) taking turns, and (3) staying on task.

### Minimizing Disruption

All participants spoke about, and we observed, how the mute button influenced the way they interacted in the postpartum group. Most participants remained muted when they were not talking. Reasons for this included not wanting the noises of their babies to be heard as this might make hearing conversations difficult. For example, one participant stated:

I use it (mute button) to have some respect. So, in case I know my girls [twin babies] would be crying or trying to burp them […] I know that if somebody else is speaking I want to be able to hear them and I want other people in the group to hear them so I can just mute myself so my background noise isn’t interfering with their input so I’m very comfortable using it and I appreciate it when other people use it. (P4)

In this case, the participant is describing using the mute button to demonstrate respect. This participant believed that the mute button could be used to minimize background noise out of respect for others. While there were sporadic and brief periods when a few participants remained unmuted, the PI and RC observed that the practice of using the mute button was discursively reinforced by most participants. The mute button was an integral part of how participants engaged.

The norm of remaining muted has particular implications for the postpartum virtual environment with respect to the sound of a baby’s cries or background noise. This type of silencing is interesting when compared to in-person support groups where participants would fully hear other babies and parents’ voices. During an in-person meeting, one might leave the room if their baby was noisy, but online, participants said they leveraged the mute function to minimize disruption. Several participants mentioned that this practice was derived from Zoom etiquette they developed in professional or educational settings, where muting to reduce disruptions was expected. For example, one participant noted: “I’ve always been taught in professional Zoom or anything, when you log in you mute yourself until you speak” (P32). The different intent and character of a professional meeting as opposed to a postpartum support setting are noteworthy. Yet, it appears that professional Zoom etiquette is a hegemonic discourse, and its associated practices, including muting, have infiltrated personal spaces, requiring parents to possibly eliminate the noise of their baby and other children.

All participants were somewhat concerned with the noises they made, but the noises of their babies were a significant consideration. They felt they should mute the noise of their babies (or other children) to not interrupt others or overtake their audio, a practice not possible in person. For example, one participant said that they “didn’t want her [baby] to squeal over everyone” (P5). Participants also noted that not using the mute button could be disrespectful, distracting, frustrating, annoying, and embarrassing. For example, a participant described a “weird fear of being that person that goes into the meeting unmuted and then something weird happens or I say something, just being the one that you can hear in the background” (P29). Similarly, P36 mentioned “overthinking” the mute button during the session. These words demonstrate the intense feelings about being unintentionally heard while participating in postpartum virtual support.

Some participants said that the mute button reduced uncomfortable feelings about having loud babies/children. One parent said:

I love the mute button, it makes it you have zero bad feelings about the level of noise your child is or is not making and not having to step out of the room […] I could be listening at the same time. (P22)

This may relate to discourses of children being seen, not heard, and feeling like a bad parent if your baby is crying. The Zoom etiquette of muting helped this participant navigate the discourse of kids needing to be quiet, while also reflecting the agency the parents have in terms of controlling what is heard and how they can listen. Further, the mute button enabled participants to multi-task, allowing them to attend to their children, for example, while continuing to be “online,” an engagement that may not be possible if this participant had to physically leave an in-person room. The presence and affordances of a mute button led to the development of a particular set of practices related to online communication and Zoom etiquette.

### Taking Turns

The affordances of Zoom technologies and the desire to use them “professionally” meant that participants experienced conversation on Zoom as fragmented as they felt they had to take turns in an orderly fashion. The flow of communication is disrupted as there is often a pause before someone unmutes and starts speaking:

I thought I wonder if I should leave myself unmuted for more natural flow of conversation, there’s that pause when you go to unmute and then I think sometimes it can sort of break up the flow of a conversation. But I was more concerned that if I left myself unmuted the background noise of [baby] doing whatever he was doing was going to cut out and impact the audio quality for everybody. (P14)

In this case, the expectations of appropriate Zoom etiquette outweighed the desire to have a free-flowing conversation. The participant valued not interrupting, hearing others, and being heard over a more natural flow. She prioritized Zoom etiquette and chose to use her mute button for the whole support session, again exemplifying agency in choosing how she leveraged technological affordances to participate. We see how the meaning she created about online communication was influenced by norms in the virtual environment as well as her expectations about how people should communicate.

Participants discussed the unspoken but widely understood etiquette about how to participate and communicate virtually. For example, one participant said how:

Everyone kind of was on the same mindset of Zoom call etiquette where […] for the most part it would be like one of us spoke until almost all or all of us had our opinion or our feelings stated […] I know we never discussed that, it’s just something we all naturally did. (P6)

In this instance, P6 describes how ingrained Zoom etiquette was for her and her group. Another participant said that online, “you don’t really unmute yourself and then talk when they’re talking […] there’s just like an etiquette to Zoom that’s not really present in-person per se” (P32), exemplifying how remaining muted was part of Zoom etiquette.

The muting capability of Zoom was an active contributor to restructuring communication. It worked against the back-and-forth nature of in-person conversations, minimizing informal verbal affirmations and other auditory indicators of engagement. For example, many participants noted that they did not want to unmute just to agree with someone and then re-mute, they did not want to interrupt, and they wanted to give everyone their space/turn to talk. They discussed refraining from providing supportive, responsive, or casual verbal agreement that they might do in person because they did not wish to turn off their mute button. Participants would only unmute and speak if they had a substantial point to add, serving to formalize the nature of the online communication. P21 noted that had the session been in person, she would have agreed with others or remarked how she had similar experiences but refrained from doing this at all while virtual. This fragmentation meant that participants may experience the virtual environment as less supportive/encouraging than an in-person environment. Although we had reminded participants that they could use the chat space or reaction buttons, the PI and RC observed that most did not use these. As this was a postpartum gathering and most participants had their babies with them, the physical act of typing might also have been difficult.

These practices of pausing and only speaking if adding something significant led to another important feature of Zoom etiquette: turn-taking. Each participant would wait until everyone had a chance to unmute, speak, and re-mute to answer a facilitation question before moving on. Further, we observed that if someone had their hands busy with their baby, other children, etc., it might make it harder to use the software to unmute and, by extension, to be heard and be a part of the conversation.

While there were challenges, there were benefits associated with this organized manner of communicating. The “turn-taking behavior” required by Zoom meant that people who might find it challenging to “jump into” a conversation in person can find it easier to participate virtually. Certainly, some participants missed the free-flowing conversation in person; however, some noted that the unspoken Zoom etiquette, wherein everyone gets their own space to talk, interruption free, was helpful for them. P7 discussed how the slow, turn-taking culture of the group made her feel included. Similarly, one participant noted:

I’m more of an introverted, shy or quiet person […] I find I’m thrown out by people who talk a lot more generally sometimes, so it’s nice to have that space to really be able to share and have a designated time where I could talk. (P27)

Another participant noted how “people took the same number of turns and whoever was talking got to completely finish and stop and no one interrupts and you can’t really interrupt someone which is probably very beneficial for certain personalities” (P22). The affordances of Zoom and the discursive inscription of these affordances in the form of etiquette worked together to create inclusion of more introverted participants in a postpartum support group.

### Staying on Task

Most participants mentioned missing the “side conversations” often present at in-person gatherings. They noted that when meeting people virtually, there is no way to talk with another parent one-on-one, which is a helpful way to make meaningful, longer-lasting connections with others, and a vital part of postpartum support. As one participant explained:

I think it’s awkward when you’re in a Zoom group if you’re feeling that connection with someone and being “hey everybody else who is in this call just ignore what I’m talking about for five seconds but mom over here do you want to grab a coffee sometime?” Like it just doesn’t feel right to do something like that. (P14)

Feeling unable to individually connect with other participants demonstrated how conventional Zoom etiquette prioritized staying “on task” and focusing on the full group discussion. P14 wanted to connect further with someone but in the end, refrained from doing so because Zoom felt like an inappropriate space for this. This perspective reinforces the discourse of professionalism which has been constructed in Zoom spaces.

Discourses related to the professional intent of Zoom are built into our collective consciousness. We receive an invitation to a Zoom “meeting,” and the meeting is scheduled with an official start and end time, controlled by the host. The ways in which the host chooses to leverage this Zoom capability influence whether there is space or time for informal conversation before or after the official start time. Participants may also be unaware of the “schedule” during the Zoom meeting, if it exists, whereas the host is aware of this, creating uncertainty in terms of length of time for discussions or sharing about a certain topic for participants before having to “move on.”

While participants may have used the private messaging tool to contact others through Chat function (the research team would have no way to know if this happened), there was little use of the public Chat for such connections, and no one described taking this approach during interviews. This is noteworthy because participants described hoping to forge informal connections with other parents, as they believed it would allow them to form deeper connections with other parents that might extend beyond the group into friendships. This did not happen during the formal Zoom sessions. The absence of side conversations exemplified how the Zoom platform encouraged certain types of conversations (formal, on-task), while actively discouraging others (informal, off-task). Zoom does not easily facilitate the types of connections that parents can experience in person and that they value.

In many instances, participants reflected on missing the embodied nature of face-to-face conversations and being in the physical presence of another person. We noted among our participants a “longing” for physical interaction. For example, one participant noted that they missed the opportunity for individual connection:

I think when you’re in person, without distancing restrictions […] you can make little side conversations with people, kind of get to know people that way as well as whatever general conversation is going on. And that’s tricky to do on a virtual platform. (P12)

Some participants mentioned the possibility of private messaging, although they had not used that approach, or had other ideas to make up for the lack of side conversations, such as sharing contact information or having a Facebook group.

It was evident in our study that the Zoom platform was able to easily support a certain kind of conversation and connection that was helpful; however, the materiality of Zoom’s mute function did not encourage spontaneous, casual, and relaxed conversations that are often desired between parents during the postpartum period. Important connection pieces appear to be missing due to how Zoom structures conversations. Participants described how side conversations are an important part of postpartum support that the virtual environment has not been able to facilitate. The meaning of side conversations can be related to why people come to these groups: for support, connection, and information from others.

## Discussion

Videoconferencing is an efficient and sometimes accessible way for people to connect through technology when they are physically apart. We were interested in learning more about how people, tools, and spaces come together to structure postpartum information and support in videoconferenced environments, as well as how these practices are personally constructed within wider discourses of parenthood. Sociomaterialism and FPS allowed us to carefully analyze these tools and practices, where we found that the looming presence of the mute button and the etiquette it constructs, including minimizing disruptions, turn-taking behaviors, and streamlined, on-task conversations, meant that Zoom technology actively shapes communication in postpartum support groups. The technology added extra steps or barriers to the act of making a verbal or auditory comment. It also creates work for participants, as they must weigh the pros and cons and decide whether their words are worth the effort of unmuting and engaging.

As we collected observational data, and through our interviews with participants, we learned that the material realities of Zoom were serving as “forcible mediators of communication” (Lögdlund, 2010, p. 183). In videoconferenced postpartum sessions, the social connections that participants had hoped to establish were woven together with the materiality of the Zoom technologies. The material realities of the videoconferencing tools—the mute button, the microphone, and the chat feature, among others—come together in a way that made opting to be silent, avoiding making spontaneous comments, or never changing the purpose of the conversation at hand the best way to be in online spaces. These principles are discursively inscribed as the required Zoom etiquette, and there is little room to function outside of it. This etiquette, in turn, shapes the quality and flow of the communication in the online space, complicating any activity that might be perceived as a distraction from the promise of seamlessly connecting in virtual spaces.

Aligned with our findings, some studies have noted how Zoom can disrupt the flow or “rhythm” of conversation (Balters et al., 2023; Boland et al., 2022). Specifically, one study found that participants on Zoom took fewer but longer turns to speak, which is congruent with our participants’ comments on only speaking if they have something substantial to add to the conversation (Boland et al., 2022). However, this was not attributed to the mute button, as studies did not use the mute button and conversations were conducted in one-on-one, controlled environments. Conversely, university instructors noted that since students were asked to mute when not speaking, they had less opportunity to provide spontaneous reactions to jokes or comical situations (Mordechai, 2020). Instructors also reflected on how the practice of staying behind after a lesson (or arriving early) to ask further questions or chat to fellow students was non-existent on Zoom, which aligns with our findings on how Zoom does not allow for side conversations and thus curtails connection building before or after the support session’s official start and end time. Although the classroom setting is different from the postpartum group support context, both fields of research could learn from each other about communication and connection in the virtual environment.

Participants described longing to be in the same physical space, or presence, of other participants. We noticed multiple understandings at play in participants’ hopes for connection, many of which relate to conventional embodied perceptions of “space.” Traditionally, space was thought to be concrete (facilities, technical artifacts, and bodies), and participants sentimentally described a utopian vision of being together in a shared space. This is not surprising given the context of a global pandemic; however, as digital technologies have advanced, the realities of space have evolved. In this digital age, space may also be considered a social construct, meaning a set of relations between people within cognitive and virtual realms (Lögdlund, 2010; Soja, 1985).

Our research examined how people and material objects—in our case, parents and Zoom technologies—come together to generate a platform for intimacy and social connection. Even before the COVID-19 era, a burgeoning literature described the affordances of such technologies to build connections. For example, Buse and colleagues (2018) discussed the “materialities of care,” while Lupton (2019) described how deeply held assumptions about space, time, and materiality affected both formal and informal healthcare experiences. Literature exploring the search for technologically mediated “mundane intimacies” (Hjorth et al., 2018) and their related “intimate entanglements” (Latimer & López Gómez, 2019) drew attention to how social connection, in any form, is a sociomaterial assemblage. In other words, social connections are dynamically configured with and through material objects such as the mute button, the device, or the screen.

Watson and colleagues (2021) noted that the literature on digitally mediated intimacies consistently points to an important opportunity for technologies, like Zoom, to facilitate affection, friendship, familial ties, emotional connection, and concern for others. Yet, we found our participants longing for a physical presence. Sociomaterial approaches have noted that focusing on the behaviors required through such technologies can surface why they continue to be less than optimally activated. Pols (2012) explored how devices are used to facilitate telehealth communications and noted that the experience of “care at a distance” was experienced as “cold” in contrast to in-person interactions, which felt “warm.” However, other studies have shown how relationships can be built and sustained in digital environments, including through social media platforms (Farci et al., 2017; Madianou, 2016), and that already established relationships, including with family members, can be nurtured through digital tools (Longhurst, 2016; Madianou, 2016).

Our work is the first to focus on the agency of the mute button in controlling intimacy and sociality in the digital postpartum environment. This focus on “mute” advances the academic discussion. For example, Watson and colleagues (2021) noted the centrality of video connections through the camera to develop a co-presence (Hjorth et al., 2015) and allow for a feeling of warmth and closeness. In contrast, our work demonstrates that the mute button, when used outside of professional contexts, may actively work against feelings of connection.

Our findings reinforce Fenwick’s (2014) assertion that “materials act together with other elements and forces (discourses, symbols, desires, etc.) to exclude, invite, and regulate particular forms of participation” (p. 269). While participating in videoconferenced postpartum support and information sessions can spark an interest in forging a deeper connection, it appears that the mute button is the source of significant tensions and that feelings of connection might occur in spite of, rather than because of, Zoom technologies. The microphones and relatedly the mute button become an obligatory point of passage (Callon, 1986), through which all actors in the postpartum group setting must flow. The materiality of Zoom therefore shapes group sessions in ways that are, at first glance, easy to dismiss as unimportant. However, when we deliberately attune to these features, we see that they actively work against generating the feeling of warmth and closeness people seek, especially during the postpartum period where loneliness is a commonly shared experience. This is not to suggest that there is no value in connecting using Zoom (or other similar platforms) during the postpartum era. Likewise, we are not espousing having microphones on all the time. As our participants noted, there is value in using the mute button. We encourage organizers of postpartum virtual support sessions to acknowledge the below considerations.

### Implications for Practice

1. Zoom (and other videoconferencing platforms) can contribute to disruption because they amplify sound, which is in part why constant use of the mute button is common. As facilitators and researchers, we recognized that the mute button controls conversations, where participants felt pressure to use the mute button for respect and focus. Facilitators should talk about the mute button before sessions begin, discussing the ways in which the group might use mute buttons to minimize distractions, allow conversations, focus, etc.
2. Zoom creates an environment where conversations are fragmented, and it is difficult to casually agree with someone. Participants did not want to turn on their microphones unless they had a substantial point to add, resulting in fewer supportive or understanding verbal agreements. Facilitators should acknowledge this difficulty and make it clear that it is important to reassure each other in small ways, perhaps by using the chat bar and reaction emojis, or turning on the microphone and speaking up, even if only to agree with someone.
3. As the meeting is controlled by the host and the discussion can only occur in a large group, people miss side conversations where they can form a deeper connection with one or more other participants. Facilitators should come up with creative ways for participants to have side conversations such as encouraging the chat space, sharing contact information, or leaving the meeting open for 5 minutes after the session is “officially” over.

### Strengths and Limitations

Other research has shown that, no matter the method or platform, new parents want to form connections and seek out information and support from one another (Aston et al., 2021; Price et al., 2018). This was the first study to our knowledge to explore the videoconferencing environment as a tool for postpartum support and information while examining the material and social aspects of the virtual environment. As such, this study unveils important information on how parents may facilitate connection in the virtual environment.

Participants in this study had to have access to Zoom via laptops, tablets, and/or cell phones; thus, this study by nature was exclusive to parents and guardians who did not have access to these electronic devices and/or stable Wi-Fi/internet connection. Our participants were predominantly women, mothers, White, in middle-to-upper socioeconomic positions, and heterosexual, therefore lacking in diversity. Future research could build upon our findings and include more diversity.

## Conclusion

These findings help to illuminate how new parents experience the videoconferencing environment in the context of postpartum support and information. Using both sociomaterialism and FPS as a means of analysis, we learned that the mute button was a discursive practice within the broader discourse of Zoom etiquette developed by professionalism in the virtual environment. Most participants used the mute button during the length of postpartum support sessions unless speaking, which structured conversation by minimizing disruptions, impeding the flow of conversations, encouraging turn-taking, discouraging small comments, and inhibiting one-on-one connections by ensuring the group stays on task. Facilitators of virtual postpartum support groups should consider audio disruptions and how the mute button controls conversation, encourage participants to reassure one another through small ways, and use creative means to ensure participants are still able to make one-on-one connections with one another. We must be thoughtful and deliberate in terms of facilitating and delivering postpartum support and information in online environments.

## References

[bibr1-10497323231187541] Adams S. (2020, April 22). Zoom meeting etiquette: 15 tips and best practices for online video conference meetings. Pennlive. https://www.pennlive.com/coronavirus/2020/04/zoom-meeting-etiquette-15-tips-and-best-practices-for-online-video-conference-meetings.html

[bibr2-10497323231187541] Alang S. M. Fomotar M. (2015). Postpartum depression in an online community of lesbian mothers: Implications for clinical practice. Journal of Gay & Lesbian Mental Health, 19(1), 21–39. 10.1080/19359705.2014.910853

[bibr3-10497323231187541] Andersen C. H. (2021, January 6). 14 Zoom etiquette rules you need to follow. Reader’s digest. https://www.rd.com/article/zoom-etiquette/

[bibr4-10497323231187541] Archibald M. M. Ambagtsheer R. C. Casey M. G. Lawless M. (2019). Using Zoom videoconferencing for qualitative data collection: Perceptions and experiences of researchers and participants. International Journal of Qualitative Methods, 18, 1609406919874596. 10.1177/1609406919874596

[bibr5-10497323231187541] Aston M. (2016). Teaching feminist poststructuralism: Founding scholars still relevant today. Creative Education, 07(15), 2251–2267. 10.4236/ce.2016.715220

[bibr6-10497323231187541] Aston M. Price S. Etowa J. Vukic A. Young L. Hart C. MacLeod E. Randel P. (2014). Universal and targeted early home visiting: Perspectives of public health nurses, managers and mothers. Nursing Reports, 4(1), 12–18. https://dx.doi.org.ezproxy.library.dal.ca/10.4081/nursrep.2014.3290

[bibr7-10497323231187541] Aston M. Price S. Hunter A. Sim M. Etowa J. Monaghan J. Paynter M. (2021). Second opinions: Negotiating agency in online mothering forums. Canadian Journal of Nursing Research, 53(4), 327–339. 10.1177/0844562120940554 32757828

[bibr8-10497323231187541] Aston M. Price S. Monaghan J. Sim M. Hunter A. Little V. (2018). Navigating and negotiating information and support: Experiences of first-time mothers. Journal of Clinical Nursing, 27(3–4), 640–649. 10.1111/jocn.13970 28722771

[bibr9-10497323231187541] Balters S. Miller J. G. Li R. Hawthorne G. Reiss A. L. (2023). Virtual (Zoom) Interactions alter conversational behavior and interbrain coherence. Journal of Neuroscience, 43(14), 2568–2578. 10.1523/JNEUROSCI.1401-22.2023 36868852 PMC10082458

[bibr10-10497323231187541] Beauford M. (2022). The state of video conferencing in 2022. GetVoIP. https://getvoip.com/blog/2020/07/07/video-conferencing-stats/

[bibr11-10497323231187541] Boitnott J. (2021). 8 Zoom etiquette rules everyone should follow. Entrepreneur. https://www.entrepreneur.com/article/383772

[bibr12-10497323231187541] Boland J. E. Fonseca P. Mermelstein I. Williamson M. (2022). Zoom disrupts the rhythm of conversation. Journal of Experimental Psychology: General, 151(6), 1272–1282. 10.1037/xge0001150 34748361

[bibr13-10497323231187541] Boudiaf L. Dupont F. Gras-Le Guen C. Sauvaget A. Leroy M. Thubert T. Winer N. Dochez V. Link to external site, this link will open in a new window (2022). Postpartum depression after maternal isolation during the COVID-19 Pandemic: The MUMI-19 study (Mothers Undergoing Mental Impact of COVID-19 Pandemic). Journal of Clinical Medicine, 11(19), 5504. 10.3390/jcm11195504 36233372 PMC9573123

[bibr14-10497323231187541] Buse C. Martin D. Nettleton S. (2018). Conceptualising ‘materialities of care’: Making visible mundane material culture in health and social care contexts. Sociology of Health & Illness, 40(2), 243–255. 10.1111/1467-9566.12663 29464775

[bibr15-10497323231187541] Callon M. (1986). Elements of a sociology of translation: Domestication of the scallops and the fishermen of St Brieuc Bay. In Power, action, and belief: A new sociology of knowledge? (pp. 196–233). Routledge.

[bibr16-10497323231187541] Callon M. Rip A. Law J. (1986). Mapping the dynamics of science and technology: Sociology of science in the real world. Springer.

[bibr17-10497323231187541] Cheek J. (2000). Postmodern and poststructural approaches to nursing research. Sage Publications, Inc. 10.4135/9781452204895

[bibr18-10497323231187541] Denton L. K. Creeley C. E. Stavola B. Hall K. Foltz B. D. (2020). An analysis of online pregnancy message boards: Mother-to-mother advice on medication use. Women and Birth, 33(1), e48–e58. 10.1016/j.wombi.2018.12.003 30545755

[bibr19-10497323231187541] Domb S. Manly E. Elman D. (2021). Pandemic patch-up: Using Zoom^TM^ videoconferencing software to create a virtual teaching clinic. Canadian Family Physician, 67(1), 65–68. 10.46747/cfp.670165 33483398 PMC7822599

[bibr20-10497323231187541] Farci M. Rossi L. Boccia Artieri G. Giglietto F. (2017). Networked intimacy. Intimacy and friendship among Italian Facebook users. Information, Communication & Society, 20(5), 784–801. 10.1080/1369118X.2016.1203970

[bibr21-10497323231187541] Fenwick T. (2014). Knowledge circulations in inter-para/professional practice: A sociomaterial enquiry. Journal of Vocational Education & Training, 66(3), 264–280. 10.1080/13636820.2014.917695

[bibr56-10497323231187541] Foucault M. (1982). The Subject and Power. Critical Inquiry, 8(4), 777–795. http://www.jstor.org/stable/1343197

[bibr22-10497323231187541] Gilbert S. (2021, March 10). Becoming a parent during the pandemic was the hardest thing I’ve ever done. The Atlantic. https://www.theatlantic.com/culture/archive/2021/03/isolation-becoming-new-parent-during-pandemic/618244/

[bibr23-10497323231187541] Hjorth L. Horst H. Pink S. (2015). Digital kinships: Intergenerational locative media in Tokyo, Shanghai, and Melbourne. In Routledge handbook of new media in Asia (pp. 251–262). Routledge.

[bibr24-10497323231187541] Hjorth L. Pink S. Horst H. (2018). Being at home with privacy: Privacy and mundane intimacy through same-sex locative media practices. International Journal of Communication, 12(2018), 1209–1227.

[bibr26-10497323231187541] Jackson L. De Pascalis L. Harrold J. A. Fallon V. Silverio S. A. (2021). Postpartum women’s psychological experiences during the COVID-19 pandemic: A modified recurrent cross-sectional thematic analysis. BMC Pregnancy and Childbirth, 21(1), 625. 10.1186/s12884-021-04071-2 34530772 PMC8445650

[bibr27-10497323231187541] Jago C. A. Singh S. S. Moretti F. (2020). Coronavirus disease 2019 (COVID-19) and pregnancy: Combating isolation to improve outcomes. Obstetrics and Gynecology, 136(1), 33–36. 10.1097/AOG.0000000000003946 32384386

[bibr28-10497323231187541] Joy P. Aston M. Price S. Sim M. Ollivier R. Benoit B. Akbari-Nassaji N. Iduye D. (2020). Blessings and curses: Exploring the experiences of new mothers during the COVID-19 pandemic. Nursing Reports, 10(2), 207–219. Article 2. 10.3390/nursrep10020023 34968364 PMC8608056

[bibr29-10497323231187541] Kynø N. M. Fugelseth D. Knudsen L. M. M. Tandberg B. S. (2021). Starting parenting in isolation a qualitative user-initiated study of parents’ experiences with hospitalization in neonatal intensive care units during the COVID-19 pandemic. PLoS One, 16(10), e0258358. 10.1371/journal.pone.0258358 34714832 PMC8555791

[bibr30-10497323231187541] Latimer J. López Gómez D. (2019). Intimate entanglements: Affects, more-than-human intimacies and the politics of relations in science and technology. The Sociological Review, 67(2), 247–263. 10.1177/0038026119831623

[bibr31-10497323231187541] Lee K. Vasileiou K. Barnett J. (2019). “Lonely within the mother”: An exploratory study of first-time mothers’ experiences of loneliness. Journal of Health Psychology, 24(10), 1334–1344. 10.1177/1359105317723451 28795604

[bibr32-10497323231187541] Lögdlund U. (2010). Constructing learning spaces? Videoconferencing at local learning centres in Sweden. Studies in Continuing Education, 32(3), 183–199. 10.1080/0158037X.2010.517993

[bibr33-10497323231187541] Longhurst R. (2016). Mothering, digital media and emotional geographies in Hamilton, Aotearoa New Zealand. Social & Cultural Geography, 17(1), 120–139. 10.1080/14649365.2015.1059477

[bibr34-10497323231187541] Lupton D. (2019). Toward a more-than-human analysis of digital health: Inspirations from feminist new materialism. Qualitative Health Research, 29(14), 1998–2009. 10.1177/1049732319833368 30964392

[bibr35-10497323231187541] MacLeod A. Ajjawi R. (2020). Thinking sociomaterially: Why matter matters in medical education. Academic Medicine: Journal of the Association of American Medical Colleges, 95(6), 851–855. 10.1097/ACM.0000000000003143 31876568

[bibr36-10497323231187541] MacLeod A. Cameron P. Kits O. Power G. Tummons J. (2019). Teaching and learning with videoconferencing at regional medical campuses: Lessons from an ethnographic study. Journal of Regional Medical Campuses, 2(2). Article 2. 10.24926/jrmc.v2i2.1559

[bibr37-10497323231187541] MacLeod A. Kits O. Whelan E. Fournier C. Wilson K. Power G. Mann K. Tummons J. Brown P. A. (2015). Sociomateriality: A theoretical framework for studying distributed medical education. Academic Medicine, 90(11), 1451–1456. 10.1097/ACM.0000000000000708 25830536

[bibr38-10497323231187541] Madianou M. (2016). Ambient co-presence: Transnational family practices in polymedia environments. Global Networks, 16(2), 183–201. 10.1111/glob.12105

[bibr39-10497323231187541] McLaren L. (2018). The excruciating loneliness of being a new mother. Today’s Parent. https://www.todaysparent.com/baby/postpartum-care/the-excruciating-loneliness-of-being-a-new-mother/

[bibr40-10497323231187541] Mordechai G. (2020). Synchronous teaching and learning: On-ground versus Zoom. International Journal of Education and Human Developments, 6(3). 11–19.

[bibr41-10497323231187541] Nowland R. Thomson G. McNally L. Smith T. Whittaker K. (2021). Experiencing loneliness in parenthood: A scoping review. Perspectives in Public Health, 141(4), 214–225. 10.1177/17579139211018243 34286652 PMC8580382

[bibr42-10497323231187541] Ollivier R. Aston D. M. Price D. S. Sim D. M. Benoit D. B. Joy D. P. Iduye D. Nassaji N. A. (2021). Mental health & parental concerns during COVID-19: The experiences of new mothers amidst social isolation. Midwifery, 94, 102902. 10.1016/j.midw.2020.102902 33421662 PMC9756383

[bibr43-10497323231187541] Pols J. (2012). Care at a distance: On the closeness of technology (1st ed., Vol. 13). Amsterdam University Press.

[bibr44-10497323231187541] Price S. L. Aston M. Monaghan J. Sim M. Tomblin Murphy G. Etowa J. Pickles M. Hunter A. Little V. (2018). Maternal knowing and social networks: Understanding first-time mothers’ search for information and support through online and offline social networks. Qualitative Health Research, 28(10), 1552–1563. 10.1177/1049732317748314 29281945

[bibr45-10497323231187541] Robinson A. Davis M. Hall J. Lauckner C. Anderson A. K. (2019). It takes an e-village: Supporting African American mothers in sustaining breastfeeding through Facebook communities. Journal of Human Lactation, 35(3), 569–582. Scopus. 10.1177/0890334419831652 30889373

[bibr46-10497323231187541] Soja E. W. (1985). The spatiality of social life: Towards a transformative retheorisation. In Gregory D. Urry J. (Eds.), Social relations and spatial structures (pp. 90–127). Macmillan Education UK. 10.1007/978-1-349-27935-7_6

[bibr47-10497323231187541] Stana A. Miller A. R. (2019). “Being a mom = having all the feels”: Social support in a postpartum depression online support group. Atlantic Journal of Communication, 27(5), 297–310. 10.1080/15456870.2019.1616736

[bibr48-10497323231187541] Taylor B. L. Howard L. M. Jackson K. Johnson S. Mantovani N. Nath S. Sokolova A. Y. Sweeney A. (2021). Mums alone: Exploring the role of isolation and loneliness in the narratives of women diagnosed with perinatal depression. Journal of Clinical Medicine, 10(11), 2271. 10.3390/jcm10112271 34073903 PMC8197355

[bibr49-10497323231187541] Valtchanov B. Parry D. Glover T. Mulcahy C. (2014). Neighborhood at your fingertips: Transforming community online through a Canadian social networking site for mothers. Gender, Technology and Development. 18(2), 187–217. 10.1177/0971852414529481

[bibr50-10497323231187541] Wang E. Link to external site, this link will open in a new window Gellman C. Wood E. Garvey K. L. Connolly C. Sharon B. Alison P. Abraham C. (2022). A medical student postpartum telehealth initiative during the COVID-19 pandemic. Maternal and Child Health Journal, 26(1), 65–69. 10.1007/s10995-021-03314-0 34854027 PMC8635469

[bibr55-10497323231187541] Watson A. Lupton D. Michael M. (2021). Enacting intimacy and sociality at a distance in the COVID-19 crisis: The sociomaterialities of home-based communication technologies. Media International Australia, 178(1), 136–150. 10.1177/1329878X20961568

[bibr51-10497323231187541] Weedon C. (1987). Feminist practice and poststructuralist theory*.* Basil Blackwell.

[bibr52-10497323231187541] Weedon C. (1996). Feminist practice and poststructuralist theory (2nd ed.). Wiley-Blackwell. https://www.wiley.com/en-us/Feminist+Practice+and+Poststructuralist+Theory%2C+2nd+Edition-p-9780631198253

[bibr53-10497323231187541] Wibowo A. W. A. Rahmawati B. D. Mastrisiswadi H. (2022). Video conferencing as a face-to-face online meeting app: User preference based on usability testing. Jurnal Sistem Dan Manajemen Industri, 5(2). 10.30656/jsmi.v5i2.3432

[bibr54-10497323231187541] Zoom Communications . (2019). Video meeting etiquette: 7 tips to ensure a great attendee experience. Zoom Blog. https://blog.zoom.us/video-meeting-etiquette-tips/

